# Laboratory and Field Soil Tunneling/Tubing by Subterranean Termites in Response to 2-Phenoxyethanol

**DOI:** 10.3390/insects17020227

**Published:** 2026-02-21

**Authors:** Luke Prescott, Roberto Pereira, Rebecca Baldwin, Allen Fugler, Philip Koehler

**Affiliations:** 1 Navy Environmental and Preventive Medicine Unit 7, PSC 819 BOX 67, FPO, AE 09645, USA; lprescott711@gmail.com; 2 Entomology & Nematology Department, University of Florida, Gainesville, FL 32611, USA; rpereira@ufl.edu (R.P.); baldwinr@ufl.edu (R.B.); 3 2500 Sawmill Road, #925, Santa Fe, NM 87505, USA; allen@inzecto.com

**Keywords:** Formosan termite, Eastern subterranean termite, trail pheromone mimic

## Abstract

The chemical 2-phenoxyethanol (2-PE) has been found to elicit trail following responses in subterranean termites and could have potential use as an enhancement to termite treatments. In this paper, similar glycol ethers to 2-phenoxyethanol were found to elicit significant trail following compared to the control, but they were not as effective as 2-phenoxyethanol. Significantly more shelter tubes were built over 2-phenoxyethanol treatment lines applied to vinyl tile compared to water controls. Eastern subterranean termites chose a food source with 2-PE applied to the surrounding soil over water controls in a choice test. The termites also tunneled along a soil treatment line significantly further, and with less deviation than the water controls, in sand. During field trials, there was termite activity only at 2-PE-treated detection stations beginning at 4 months after initial installation. 2-phenoxyethanol is relatively inexpensive, considered safe for the environment, and easy to mix in large batches. 2-PE is a simple and efficient way to guide termites to specific locations in the field for collection and other purposes. These factors should facilitate adoption in future pest management practices.

## Simple Summary

The glycol, 2-phenoxyethanol (2-PE), a solvent in some pen inks, eye drops, and cosmetics, is an artificial trail pheromone that termites follow and has been used to direct termite movement. Both Formosan and Eastern subterranean termites were shown to follow 2-PE treatments applied to paper, semiporous hard surfaces, and soil. Treatments of 2-PE to soil surfaces directed subterranean termites to detection stations.

## 1. Introduction

**Figure 1:**
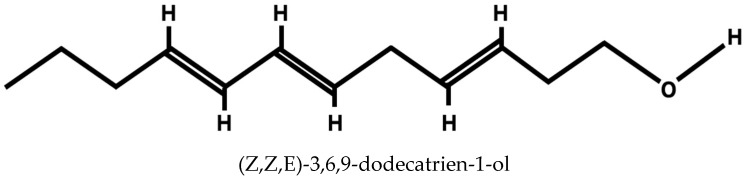
The chemical structure of the natural trail pheromone (Z,Z,E)-3,6,8-dodecatrien-1-ol of *C. formosanus* [2].

Subterranean *Reticulitermes* termites follow lines drawn with certain ballpoint ink pens, such as the brand Papermate^®^ (The Gilette Company, Boston, MA, USA) [1,2]. It was not until 1998 when the trailing compound in pen ink was isolated and identified as 2-phenoxyethanol (2-PE) using gas chromatography–mass spectrometry [2]. These glycols are known pheromone-mimicking compounds for a few termite species, including the Formosan and Eastern subterranean termites [3]. When compared to the primary trailing pheromone for *Coptotermes formosanus* and *Reticulitermes* spp., the only similar chemical compositional trait is a primary alcohol group (Figure 1) [4]. The mechanisms of action for these compounds are still relatively unknown [5].

2-phenoxyethanol (2-PE) is a benign and ecologically sound chemical [6,7]. It has industry uses which range from cosmetic preservatives to an indirect food additive and has been used in the consumer market for decades [8]. 2-PE has caused minor side effects of contact eczema and allergies in cosmetic and vaccine applications [9,10]. In 2019, the Environmental Protection Agency ruled that 2-PE was exempt from the requirement of a tolerance when it is limited to 0.2% by weight in formulations The EPA has determined that 2-PE is not a pesticide and therefore, it is not subject to the requirements of FIFRA (L. Hollis, Office of Chemical Pollution and prevention, EPA, 28 May 2024). The safety profile of 2-PE should allow it to be used to modify termite behavior near humans.

There is a need to study the behavior of *R. flavipes*, the most widespread and important termite in the Eastern U.S., in response to 2-PE and closely related glycols. Therefore, the five-fold objectives of this study are to determine the following:

- The extent of *R. flavipes* trail following in response to glycol ethers closely related to 2-PE.
- *R. flavipes* shelter tube construction over 2-PE-treated lines applied to vinyl tile (a common semiporous surface in structures).
- *R. flavipes* recruitment to soil treated with 2-PE around termite food (paper).
- The precision of *R. flavipes* in following a trail of 2-PE applied to soil.
- The effect of 2-PE in guiding *C. formosanus* to pop-up termite detection stations in field trials.

The intent of this research is to provide information that may allow us to modify termite trailing behavior in different substrates.

## 2. Materials and Methods

### 2.1. Insect Rearing and Handling

Eastern subterranean termites (*R. flavipes*) were used for the bioassays. Collected portions of in situ termite colonies (colony fragments) were taken. These fragments did not have a queen termite and were mostly termite foragers. The termite colony fragments used were taken from 4 separate colonies (one in Jacksonville, FL [Jax], one in Gainesville, FL [Solar], one in High Springs, FL {High Springs], and one in Newberry, FL {Baldwin}). Colony fragments were collected from bucket traps consisting of a bucket (2 L, 6 cm × 8 cm, Venture Packaging Inc., Monroeville, OH, USA) with holes (4 cm diameter) drilled on four sides and the bottom and was placed in a hole so that the top of the bucket was flush with the surrounding topsoil. Soil was added inside the bucket to just cover the bottom of the bucket, about 3 cm. Two cylinders of cardboard strip (Uline, Pleasant Prairie, WI, USA) were then moistened and placed into the bucket as a food source and harborage for foraging subterranean termites. The bucket lid was then placed on top, and soil was added to cover the lid and to maintain a stable temperature and humidity inside the bucket.

Termite colony fragments were moved to and maintained at the University of Florida Urban Entomology Laboratory in Gainesville, FL. Each termite colony fragment was kept in a single 13 L plastic container (Rubbermaid Co., Winchester, VA, USA, 46 × 31 × 15 cm) with five corrugated cardboard strips (Uline Co., S-1673 corrugated wrap, 30 cm × 15 cm each) stacked and placed inside for harborage and food. Colony fragments were maintained at ~24 °C and 99% RH inside the containers. Tap water was added to each container weekly via a moistened sponge (3M, St. Paul, MN, USA, 100% plant-based fibers) that was placed adjacent to the cardboard strip stack, allowing termites to access water from the sponge. The cardboard was changed every 1–2 months to ensure healthy living conditions and was structurally strong to make it easier to remove termites for bioassays. Adult workers and soldiers were used in the bioassays.

For termite collection, Tygon^®^ tubing (ID: 0.635 cm, OD: 0.952 cm, Tygon S3 E-3603, Fisher Scientific, Pittsburgh, PA, USA) with a one-way suction valve connected to a Falcon centrifuge tube (50 mL, Cat No. 06-443-21, Fisherbrand, Pittsburgh, PA, USA) was used. With the Tygon tubing connected to a vacuum port, only light suction was used to remove termites from the cardboard strips without injury. The termites were then collected in the centrifuge tube for use in experiments.

### 2.2. Chemicals

The chemicals 2-phenoxyethanol (2-PE, Acros Organics, NJ, USA, 250 mL CAS: 122-99-6), 2-propoxyethanol (Sigma-Aldrich, St. Louis, MO, USA, 1 L CAS: 2807-30-9), 2-ethoxyethanol (Sigma-Aldrich, St. Louis, MO, USA, 1 L CAS: 110-80-5), 2-butoxyethanol (Sigma-Aldrich, St. Louis, MO, USA, 1 L CAS: 111-76-2), diethylene glycol monoethyl ether (Sigma-Aldrich, St. Louis, MO, USA, 100 mL CAS: 111-90-0), and triethylene glycol (Sigma-Aldrich, St. Louis, MO, USA, 100 mL CAS: 112-27-6) were used in the bioassays. They are soluble in both aqueous and ethanol solutions (Decon Labs Inc., King of Prussia, PA, USA, 190 Proof). 2-PE is a known termite trail pheromone mimic [2], while the five other chemicals have a similar chemical structure to 2-PE and the termite trail pheromone ((Z,Z,E)-3,6,8-dodecatrien-1-ol) [1]. All are glycol ethers, with a carbon chain and a terminal alcohol.

### 2.3. Paper Trail Assay

**Figure 2:**
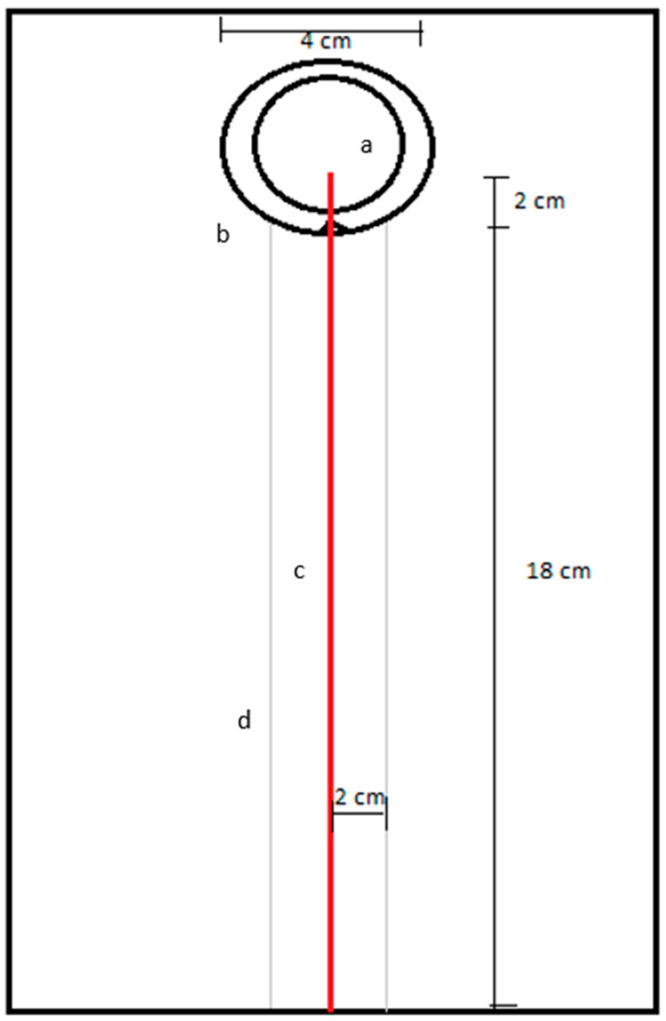
Paper trail assay on white copy paper. The smaller cup (a) contains termites while they recuperate from the transfer into the bioassay apparatus. Once the termites were recovered, the small cup was removed, and the termites could exit the large cup (b) via the triangle cut-out. The treatment trail (c) runs from the large cup center down to the bottom of the page. The barrier lines (d) were 2 cm off each side of the treatment trail and act as the boundaries to indicate when termites lost the trail. Red indicates location of treatment trail.

This experiment tested the ability of termites to follow a trail of different, but similar, chemical compounds (Figure 2). Each chemical was tested via an adaptation of the paper trail assay [11]. This was performed on a sheet of white copy paper (Office Depot, Boca Raton, FL, USA). A 20 cm straight line composed of the respective chemical at 0.08% concentration in water was applied to the paper with a 0.18 mm Rapidograph pen (Faber-Castell, Nuremberg, Germany). Each trail had an average of 3.29 mg (0.16 mg/cm) of chemical solution. After application the trail was allowed to dry for 5 min before initiation of the assay. For each replicate, a new piece of paper is used. Two plastic cups were used for the release site. A larger cup (59 mL, Dart Container Co., Mason, MI, USA) had a 1 cm × 1 cm × 1 cm triangle cut out of the lip to allow termites to enter the assay arena. A smaller cup (30 mL, Dart Container Co., Mason, MI, USA) was placed inside the larger cup. Both cups had their bottom 2 cm section removed by a circular cut via a box cutter knife. The triangle of the larger cup was facing the treatment trail and allowed 2 cm of the treatment trail to extend into the release site region. After 30 s of acclimation of the termites inside the smaller cup, this cup was removed, and the termites were able to follow the treatment trail to its completion.

For each replication, 10 termite workers randomly chosen from their respective colony fragment were used. The termites were given three minutes to follow the trail to the best of their ability. For the termites that completed the entire trail, they were recorded in the category of ‘completed trail’ as well as the time they finished at. For the termites that left the introduction site but did not complete the trail, they were categorized as ‘did not finish (DNF)’. To determine if the termite completed the trail, two straight lines, running parallel at 2 cm on either side of the treatment trail were drawn with pencil. These lines marked the buffer zone due to the serpentine nature of termite trail following. If the termites stayed inside the buffer lines and completed the 20 cm trail, those repetitions were counted as successful. If the termites at any time went outside the buffer zone lines, then they were categorized as not completing the trail and would result in a ‘DNF’.

For each chemical/colony, twenty replications (each representing 10 termites) were performed (80 total repetitions per chemical) using ten termite workers randomly chosen from their respective colony fragment container for each repetition. The response variables were the mean number of termites completing the trail, the mean time for termites to complete the treatment trail, and the average distance the termites followed the treatment trail for.

### 2.4. Shelter Tube Assay

**Figure 3:**
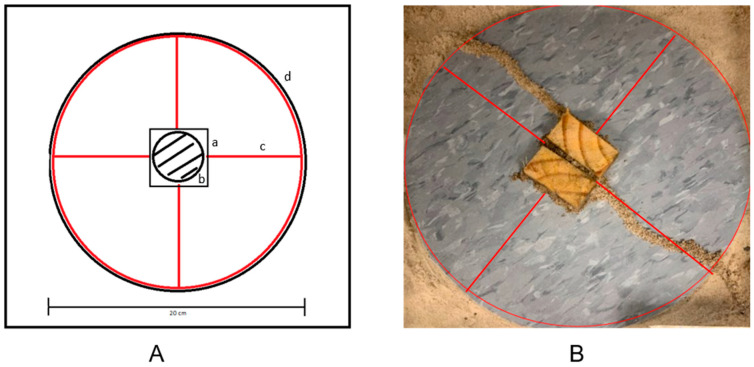
Shelter tube assay. (**A**) The tile disk (d) was placed on top of the release site (b). On the tile, a cardboard food source (a) was placed in the middle of the treatment trails shown in red (c). (**B**) Example of a treated assay result with 2-PE treatment in red..

This experiment tested the ability of termites to build foraging tubes on a 2-PE marked trail (Figure 3). A shelter tube assay was performed in a Nunc dish (Thermo Fisher Scientific, Waltham, MA, USA, Nunc A/S 24.5 cm × 24.5 cm × 2.5 cm) foraging arena. A 500 g batch of sifted paver sand with 10% water saturation was evenly hand-compacted into each foraging arena. A release site hole was made in the sand at the center of the Nunc dish using a plastic cup (120 mL, Dart Corp., Mason, MI, USA) to ensure even sizing across repetitions.

Circular pieces of vinyl tile (20 cm diameter, Standard Excelon Vinyl Composition Tile, Armstrong Commercial Tile, Lancaster, PA, USA) were pre-cut and treated with 2-phenoxyethanol (0.08% concentration in ethanol). The 2-PE was brushed (0.18 mL/cm) using a 2.54 cm paintbrush around the circumference of the tile and then across the tile to form a plus sign. For the control, only ethanol was used. After 10 min, the ethanol had evaporated. Two hundred Eastern subterranean termite workers and 2 soldiers were immediately placed into the release site, and the treated tile circles were placed into the center of the Nunc dishes and onto the surface of the sand, covering the release site. A 5 cm × 5 cm section of cardboard was then placed on the vinyl tile disk to serve as a food source target for the termites. The lid of the Nunc dish was then placed on the Nunc dish and parafilm was then wrapped around the edges to secure the lid and maintain humidity. Top-down photos (iPhone 10 XR, Apple Inc., Cupertino, CA, USA) of the arenas were taken and analyzed 72 h later.

For each of the 4 colonies, twenty Nunc dishes, each containing 200 worker termites, were used (80 total repetitions). The response variables that were measured included the total number of repetitions with shelter tubes along the 2-PE trails and the average deviation distance (mm) of shelter tubes away from 2-PE trails.

### 2.5. Food Choice Assay

**Figure 4:**
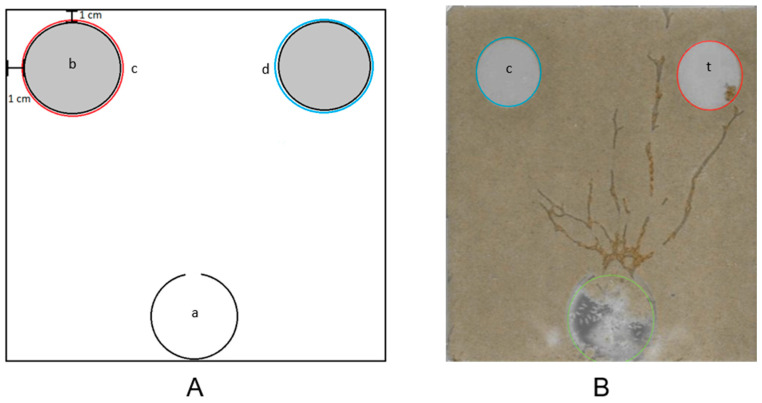
Food choice assay. (**A**) Release site (a) was located at the middle point of the bottom of the Nunc dish. The release opening faced directly toward the middle opposite side of the Nunc dish. The 2 filter paper disks (b) were in opposite corners of the release site, 1 cm from each Nunc dish wall. The 2-PE soil treatment (c) was randomly placed around one paper disk, while the water control (d) was placed around the other. (**B**) Example of an assay result. Termites tunneled to and fed on food source (t) surrounded by 2-PE treatment; food source (c) surrounded by water treatment did not receive termite tunneling or feeding. Blue indicates water treatment, red is 2-PE treatment.

This experiment tested the ability of termites to find a cellulose source surrounded by a water or 2-PE trail (Figure 4). The food choice assay was completed using Nunc dishes as foraging arenas. A plastic cup (60 mL, DartCorp., Mason, MI, USA) with the bottom cut off with a box cutter knife and triangular section (20 mm height × 10 mm base) cut out of the upper lip was flipped upside down and used as the termite release site positioned midway along one edge of the Nunc dish, with the triangular opening facing the midline of the Nunc dish. Two pre-weighed, dry, filter paper disks (42.5 mm circumference, Cat No. 1001-042, Whatman International Ltd., Maidstone, Kent, UK) were then saturated with water and placed in the two opposite corners of the Nunc dish arena away from the release site and 1 cm away from each adjacent Nunc dish wall. A 200 g batch of sifted paver sand moistened with 10% water saturation was evenly hand-compacted onto the foraging arena, on top of the filter paper, and around the release site.

A 2-phenoxyethanol solution (0.08% conc. in water) was then pipetted onto the sand around the circumference of the 2 filter paper circles at 0.18 mL/cm. For the control treatments, water was similarly pipetted. A 300 g batch of 10% water-saturated paver sand was then evenly hand-compacted on top of the treatment and around the release site.

Two hundred Eastern subterranean termite workers and two soldiers were then placed into the release site cup immediately after sand treatment. The lid was placed onto the Nunc dish arena, and the edges sealed with parafilm to maintain relative humidity.

Twenty-four hours after arena setup, the filter paper circles were dried and weighed. For each of the 4 colonies, twenty Nunc dishes, each containing 200 worker termites, were used (80 total repetitions). The response variables that were measured for this assay included initial discovery of untreated or treated paper disk, and the average consumption amount of the filter paper disks after 24 h, as measured by obtaining the filter paper disk weights using an analytical balance (ME104TE, Mettler Toledo, Columbus, OH, USA).

### 2.6. Linear Tunneling Assay

**Figure 5:**
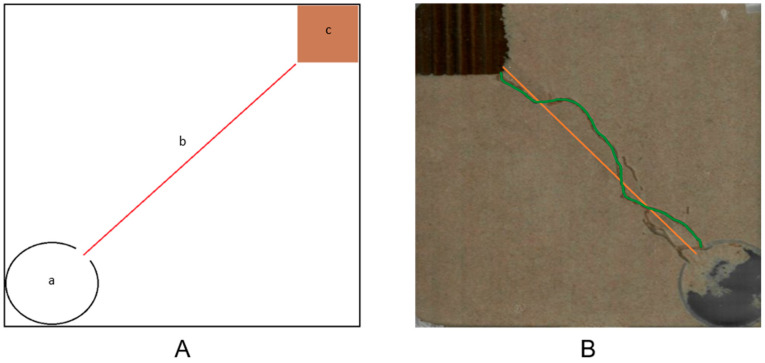
Linear tunneling assay. (**A**) The release site (a) had its opening facing towards the food cardboard food source (c). The treatment/control trail (b) ran from the opening of the release site to the food source. (**B**) Example of a treated assay result where the primary tunnel followed the treatment line. Green indicates location of temite tunnel. Red indicates location of 2-PE or water treatment.

This assay was used to test the deviation of termite tunneling from either a trail marked on sand with water or with 2-PE (Figure 5). Linear tunneling assays were completed using Nunc dishes as the foraging arena. A plastic cup (60 mL, Dart Corp., Mason, MI, USA) was used as the release site by removing the bottom half and only leaving the upper lip of the cup. A 20 mm × 10 mm-wide opening was then cut from the lip of the cup to allow termites to exit into the arena. The cut cup was then inverted so the 20 mm opening was on the floor of the Nunc dish and placed in a release corner of the dish. The 20 mm opening faced the opposite target corner of the Nunc dish. For a food foraging target, a square of corrugated cardboard (50 mm × 50 mm) presoaked in tap water was placed in the target corner of the release site. Fine paver sand (500 g; Sunniland Corp., Ocala, FL, USA) that was oven dried at 177 ± 1 °C for 2 h and strained using a 1.18 mm mesh sieve (No. 16; Thermo Fisher Scientific, Waltham, MA, USA) was used as the tunneling substrate. The sand was then separated into 300 g and 200 g batches and moistened with 10% moisture content by mixing 30 mL of water and 20 mL of water with each respective sand batch in a closed Ziploc plastic bag (3.785 L, S.C. Johnson, Racine, WI, USA). The 200 g batch of sand was then evenly hand-compacted into the Nunc dish, surrounding the release site cup and the cardboard square and covering the entire bottom of the arena.

Three milliliters of a 0.08% 2-phenoxyethanol aqueous solution was pipetted evenly at ~0.18mL/cm onto the sand to connect the release site to the cardboard food source. Control dishes were set up similarly using 3 mL of water at 0.18 mL/cm. A 300 g batch of 10% saturated sand was then hand-compacted on top of the previous sand layer and treatment/control band. Two hundred adult termite workers and two soldiers were placed into the release site cup immediately after sand treatment. The lid was added onto the Nunc dish and parafilm was used to seal the edges to maintain humidity. A bottom-view scan (using a HP Color Laser Jet Pro MFP M479fdw, HP Inc., Spring, TX, USA) of each Nunc dish was taken after 24 h to record the location and length of the primary tunnels.

For each of the 4 colonies, 40 Nunc dishes, each containing 200 worker termites, were used, 20 as control and 20 with the 2-PE treatment. Data for this assay was collected by measuring the deviation of the primary tunnel from the treatment trail using ImageJ software [12]. The primary tunnel was identified as the main tunnel that was first to find the food source, and where most termite traffic occurred. Twenty evenly spaced points were marked along the primary tunnel. Each primary tunnel length was measured and averaged.

### 2.7. Field Trials

**Figure 6:**
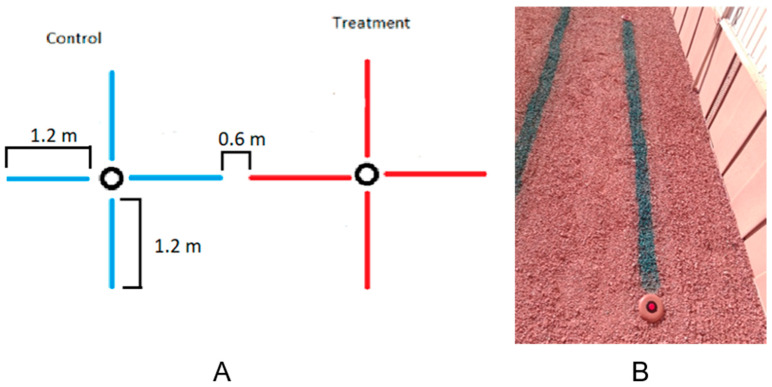
Field trial. (**A**) Control and treatment pop-up stations paired with each other. Treatment lines were 0.6 m apart and had 4, 1.2 m spray bands of either 2-PE (red) or water (blue) spray applications. (**B**) Photo shows pop-up stations installed in the ground, near a green dyed 2-PE spray band.

**Figure 7:**
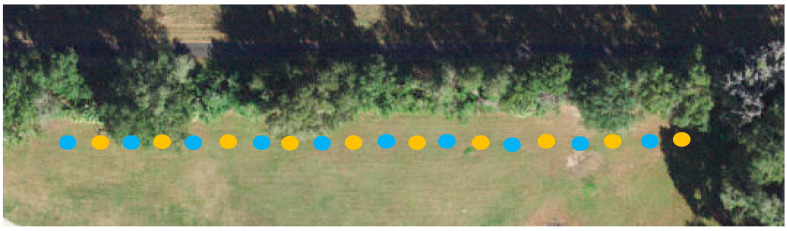
Satellite image of field trial location with blue dots representing control stations and yellow dots representing treated stations.

Field trials were completed to test the effect of 2-PE on increasing the chances of field populations of termites finding termite detection stations and shortening the time required for field populations of termites to discover termite detection stations (Figure 6 and Figure 7). Field trials of 2-PE were done on a private property in Ocala, Florida. The location previously had swarms of *C. formosanus*, providing adequate subterranean termite pressure for testing. Twenty pop-up termite detection stations (Orkin Termite Detection Program, Orkin, Walnutport, PA, USA) were used as indicators for termite foraging underground. The pop-up stations had a similar appearance to typical subterranean termite monitoring stations, but when the termites ate enough of the station’s wood, a “pop-up” mechanism was triggered that visually indicated termite activity. Along a tree line, twenty stations were placed at ~3 m intervals. The detection bait stations were placed in the ground by using a hand auger to drill holes. By using a proprietary spray nozzle, either a set of 4 control (water) or 4 treatment (0.08% 2-PE) spray bands of 1.2 m in length were sprayed at 90° angles from each other from each bait station at a flow rate of 5.5 L per ~3 linear meters on 4 February 2021. The control (water only) and treatment (2-PE) bait stations were alternated. A food-grade dye (FD&C) was added to the sprays to mark the bands of sprayed soil. There was 60 cm of unsprayed space between bait station plots.

There were 10 2-PE treated and 10 control pop-up stations. The stations were checked monthly for 6 months and rated as positive or negative for termite activity.

### 2.8. Statistical Analysis

For the paper trail assay, data on trail completion for the four tested colony fragments were arcsine square root-transformed prior to ANOVA and means were separated using Tukey–Kramer’s HSD using *p* ≤ 0.05. Data on average time to complete the trail for the four colony fragments was analyzed using ANOVA and Student’s *t*-test to compare the least-square means from each of the 6 treatments and control. Average trail following distance was analyzed using ANOVA, and means were separated using the least-squares method.

For the tube assay, mean tube deviation distance was analyzed using ANOVA, and colony means were separated using the least-squares method. Finally, a Student’s *t*-test was used to determine significant differences between the control and treatment.

For the food choice assay, a Student’s *t*-test was performed to determine significance between termites tunneling to 2-PE or to control filter paper disks. Primary tunnel lengths and primary tunnel deviation distance were separated for each colony using the least-squares method. The means for each colony were compared using Student’s *t*-test.

For the field trial work, there was no additional statistical analysis done to the data. All statistical analyses had a set significance level of α = 0.05. JMP Version 16 software [13] was used for all analyses.

## 3. Results

There was no significant difference in response between the termite colony fragments for any of the assays except for the shelter tube assay, where the Baldwin colony had a slight but significant greater deviation (16%) from the trail when compared to the other three colony fragment means (F = 8.14, df = 3, *p* < 0.0001).

### 3.1. Paper Trail Assay

Most termites did not attempt to leave the release site as they did not notice the chemical trail, unless it contained 2-PE, in which case, most of the time, the termites moved along the 2-PE treatment trail, following the path with a serpentine pattern until they reached the end of the arena. Some termites would follow the border of the release site until they found the opening and then head in a random direction on the paper, usually out of bounds. For some of the chemicals, the termites would follow the treatment trail, but take multiple pauses along the trail, as if trying to re-orient themselves. At times, the termites would leave the trail, only to turn back around to the trail.

**Figure 8:**
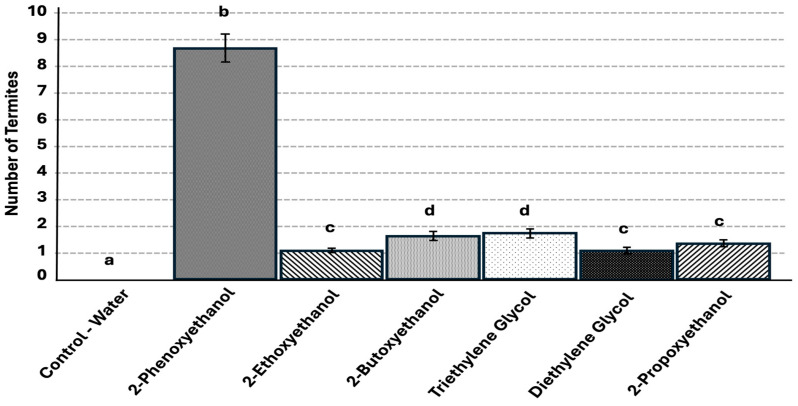
Mean number of termites per repetition (±sem) that fully completed the 20 cm treatment trail for each chemical tested (treatments not connected by the same letter are significantly different, *p* < 0.05). Error bars represent + or − std error of the mean. (N = 80 replicates per chemical [4 colonies × 20 replicates per colony]).

The completion rates were significantly greater for all tested glycol ethers compared with the control (F = 619.28, df = 6, *p* < 0.0001), for which no termites completed the trail from the release site to the end point without being eliminated according to the pre-established conditions described in the Materials and Methods Section. The 2-phenoxyethanol treatment had the highest completion rate, with 86% of termites completing the trail (Figure 8) which was significantly different from the control treatment. Triethylene glycol and 2-butoxyethanol both had a significantly lower completion rate compared to 2-phenoxyethanol at 16–17%. Of the glycol ethers, diethylene glycol monoethyl ether and 2-ethoxyethanol had the significantly lowest completion rates of ~10–11%. The control never had a termite finish the trail.

**Figure 9:**
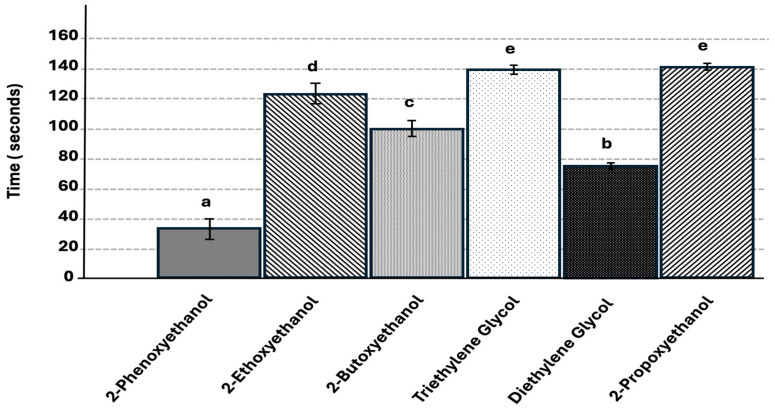
Mean time (seconds) for termites to complete the 20 cm treatment trail for each chemical tested (treatments not connected by the same letter are significantly different, *p* < 0.05). Error bars represent + or − std error of the mean. (N = 20 replicates per treatment).

The 2-phenoxyethanol treatment had the significantly lowest average completion time of 34 s (t = 1.96, *p* < 0.0001) (Figure 9). Diethylene glycol monoethyl ether had a significantly higher average time of completion in relation to 2-phenoxyethanol, at 75 s, followed by 2-butoxyethanol at 100 s, then 2-ethoxyethanol at 126 s. Of the glycol ethers, triethylene glycol and 2-propoxyethanol had the significantly highest average completion time at 140 s.

**Figure 10:**
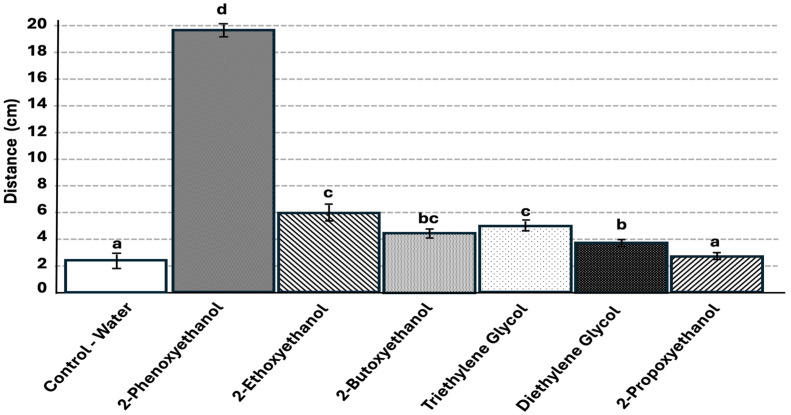
Mean distance of treatment trail (out of 20 cm total) that was followed per chemical tested. Tested treatments not connected by the same letter are significantly different, *p* < 0.05. Error bars represent + or − std error of the mean. N = 80 replicates per treatment [4 colonies × 20 replicates per colony].

The average total trail following distances were significantly greater for all tested glycol ethers compared with the control, except 2-propoxyethanol (F = 746.5, df = 6, *p* < 0.0001). The 2-phenoxyethanol treatment had the significantly greatest average total trail following distance of 19.5 cm (t = 1.96, *p* < 0.0001) (Figure 10). 2-Ethoxyethanol and triethylene glycol had significantly lower average trail following distances compared to 2-phenoxyethanol, at 5.5 cm, followed by 2-butoxyethanol at 4.4 cm, and then triethylene glycol at 3.8 cm. Of the glycol ethers, 2-propoxyethanol had the significantly lowest total trail following distance of 2.2 cm and was not significantly different from the control, which had an average total trail following distance of 2.1 cm.

### 3.2. Shelter Tube Assay

For this assay, termites would generally tunnel through the sand in a random radial pattern outward from the release site, and the tunnels would eventually come to the surface where termites would sense the 2-PE on the tile and subsequently follow the treatment trail to the food. Additional termite workers were recruited to build a shelter tube that connected the tunnel surface hole to the food source.

**Figure 11:**
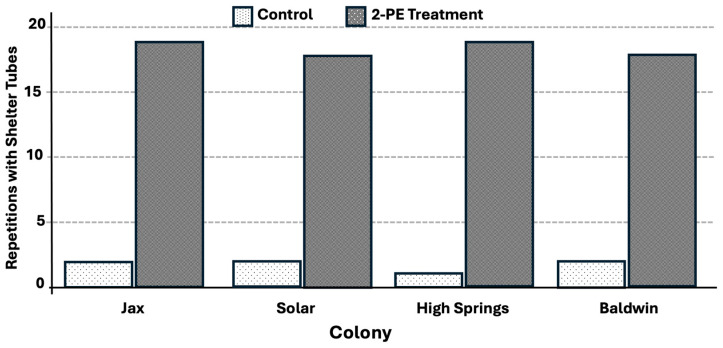
Number of repetitions (out of 20 per colony) with shelter tubes present on 2-phenoxyethanol treated trails compared with the control. All treatments were significantly different from the corresponding control.

**Figure 12:**
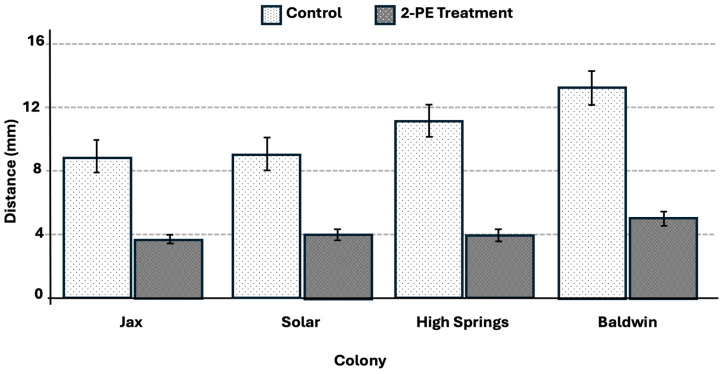
Average deviation distance of shelter tubes away from treatment trails. All treatments were significantly different from the corresponding control, *p* < 0.05. Error bars represent + or − std error of the mean.

The shelter tube formation rate was significantly greater for the 2-phenoxyethanol treatment (92.5%, t = 1.97, *p* < 0.0001) (F = 213.73, df = 1, *p* < 0.0001) (Figure 11) compared to tube formation rate of 8% in the water control. The shelter tube deviation distance was significantly lower for all treatments when compared to the control trails (Figure 12).

### 3.3. Food Choice Assay

For this assay, termites would generally tunnel through the sand in a more direct path towards the treated food source. Random foraging was not normally observed in these repetitions. Secondary tunnels were built along the primary tunnel where sand from the primary tunnel was deposited. Most of the workers were focused on tunnel excavation and sand removal.

**Figure 13:**
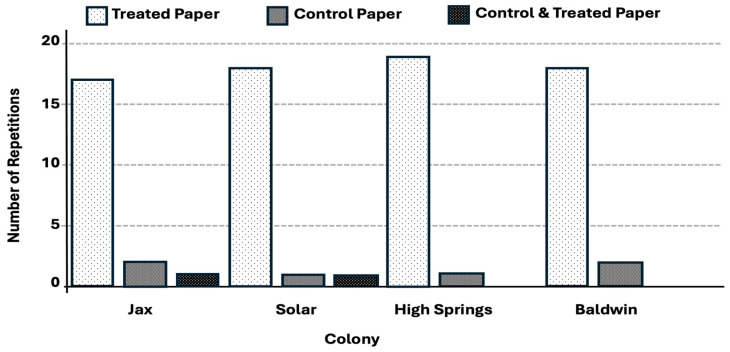
Number of repetitions in which the primary tunnel found the 2-phenoxyethanol-treated paper (T), the control paper (C), or both (TC) after 24 h.

The termites chose the 2-phenoxyethanol-treated food at a significantly greater rate (90%) than the untreated control (7.5%) (t = 1.84, *p* < 0.0001), (F = 487.3, df = 1, *p* < 0.0001) (Figure 13). For consumption of the disks, when the 2-phenoxyethanol-treated disk was chosen, an average of 7.87% of the disk weight was consumed. When the control disk was chosen, an average of 0.51% of the disk weight was consumed.

### 3.4. Linear Tunneling Assay

For this assay, termites generally tunneled in the sand along the treated trail, with the primary tunnel making a serpentine pattern, as termites would do while following 2-PE trails on paper. When first placed into the release site, most of the termites moved to the release site edge, searching for an exit. Once the release site opening was found, multiple termites started the tunneling process. During the tunneling process, multiple termites would be at the front of the tunnel, removing sand particles and passing them to other workers who would then move the particles to the release site area or to a secondary tunnel.

**Figure 14:**
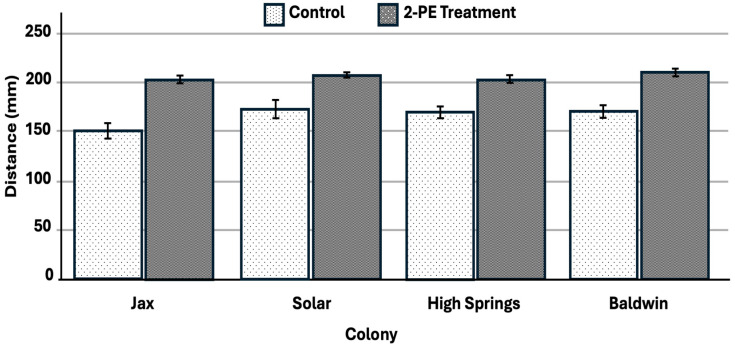
Average primary tunnel length for control and 2-phenoxyethanol repetitions after 48 h per colony fragment. Significant differences between control and treatment at *p* < 0.05 were observed for all 4 colonies. Error bars represent + or − std error of the mean.

**Figure 15:**
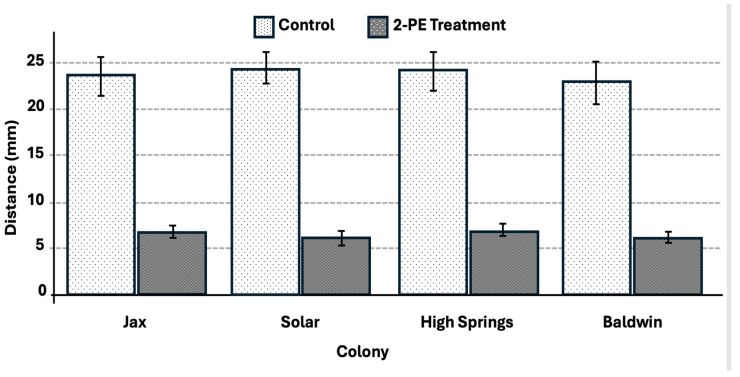
Colony fragment average for primary tunnel deviation distance in the control and 2-PE treatment trails after 48 h. Significant differences between control and treatment at *p* < 0.05 were observed for all 4 colonies. Error bars represent + or − std error of the mean.

The termites tunneled a significantly greater distance for the 2-phenoxyethanol-treated repetitions compared with the control, at an average distance of 206 mm (t = 1.975, *p* < 0.0001) (F = 111.22, df = 1, *p* < 0.0001) (Figure 14). The control had a significantly lower average primary tunnel distance of 165 mm. The average tunnel deviation distance was significantly shorter for the 2-phenoxyethanol (6.5 cm) compared with the control (23.9 cm) (t = 1.975, *p* < 0.0001), (F = 268.61, df = 1, *p* < 0.0001) (Figure 15).

### 3.5. Field Trials

For the field trials, subterranean termites in the area were able to search and find the treated stations but did not find untreated stations. Termite presence occurred at opposite ends of the field trial set up.

The treated stations were found by termites at a significantly higher rate compared with the control, with 50% of the treated stations being found within 5 months. The control stations resulted in a significantly lower rate of 0% (Table 1) after 7 months. Two treated stations were active at 3 months after treatment and an additional three stations were active at 4 months to give a total of five active stations in June.

## 4. Discussion

This study provides a comprehensive evaluation of how 2-phenoxyethanol (2-PE) and related glycol ethers influence the foraging behavior of the Eastern subterranean termite, Reticulitermes flavipes, across diverse substrates including soil, paper, and solid surfaces. Prior research has predominantly focused on the Formosan subterranean termite, *Coptotermes formosanus* [14,15], but *R. flavipes* poses the greatest threat to structures in the eastern United States [16,17]. Our findings demonstrate that 2-PE effectively mimics natural trail pheromones, overriding innate foraging patterns to guide termites along treated soil with high precision.

In the paper trail assay, termites detected and followed 2-PE lines more efficiently than other glycol ethers, exhibiting fewer pauses for antennal reorientation and completing trails in a straighter path. This behavior aligns with the role of antennation in chemical detection [18] and corroborates the 0.08% concentration as optimal for eliciting trailing behavior without toxicity [15]. As the termites followed the applied 2-PE trail, it is likely the termites would reinforce the trail with their natural trail pheromone. Our assays simulated what would occur in nature when multiple termites move along and reinforce that trail. Termites crossed untreated lines multiple times before detection, highlighting 2-PE’s superior mimicry of orientation pheromones, like (Z,Z,E)-3,6,8-dodecatrien-1-ol, which share a primary alcohol group with 2-PE but differ in volatility and persistence [4,19,20].

The shelter tube assay revealed that 2-PE directed tube construction exclusively along treated lines to food sources, causing termites to deviate from the typical fractal branching observed in untreated controls [20,21]. Tubes formed rapidly over 2-PE trails, with comparable structural integrity to controls, provided that sand moisture exceeded 10% to prevent desiccation—a critical factor for subterranean species reliant on high humidity [22,23]. Secondary tubes rarely followed 2-PE, suggesting selective recruitment signaling akin to natural pheromones from the sternal gland [24,25]. These directed termite tubes reduce exposure risks from predators, mirroring the adaptive value of shelter tubes in bridging soil nests to above-ground resources [26,27].

In the food choice assay, *R. flavipes* tunneled directly to 2-PE-treated food blocks with minimal deviation, forgoing the multiple radiating branches characteristic of exploratory foraging [28,29]. A single primary tunnel per replicate, flanked by secondary backfill paths, indicates remote detection of 2-PE in soil, similar to responses elicited by brown-rot fungi volatiles [30,31]. This overrides the energetic costs of fractal search geometries optimized for resource discovery [32] and stigmergic recruitment via recruitment pheromones [33,34], channeling effort toward the attractant.

The linear tunneling assay further showed that *R. flavipes* followed subsurface 2-PE bands in a tight serpentine pattern, excavating wider primary tunnels than in controls [31,35]. Detection thresholds appear species-specific, with *R. flavipes* responding at concentrations as low as those effective for *Hypotermes obscuriceps* (0.01%) [36], unlike higher optima for *C. formosanus* [15]. At >0.36%, 2-PE shifts to a toxicant via contact/inhalation, but sublethal doses (e.g., 0.12%) enhance consumption in choice scenarios without ethanol compounds [37].

Field trials confirmed these patterns, with termites exclusively infesting 2-PE-treated pop-up stations, though activity waned after 2 months due to limited wood resources. Untreated controls remained undiscovered, underscoring 2-PE’s potential to enhance bait station efficacy by exploiting natural multiple-nest feeding strategies [38].

These results build on foundational work showing that 2-PE extends *C. formosanus* tunneling distances and overrides dead-termite barriers when combined with non-repellent insecticides like fipronil or imidacloprid, boosting mortality via prolonged exposure [14,39]. Unlike volatile recruitment trails, 2-PE’s persistence rivals orientation pheromones lasting >1 year in *R. flavipes* [40], with low chemical diversity across termite species [18,41]. Mechanistically, 2-PE likely engages sternal gland pathways for trailing and recruitment [42,43], though quantitative species differences in blend ratios may underpin recognition [44].

Our assays reveal 2-PE as a non-native mimic that reduces search inefficiencies and energetic demands [28,45]. Compared to natural semiochemicals, like dodecatrienol and neocembrene [18], 2-PE’s glycol structure elicits analogous behaviors despite unknown receptor interactions [5], potentially via shared alcohol motifs [2]. Our research reveals a simple and efficient way to direct termite movement with 2-PE applied to soil or surfaces which may lead to practical applications. The addition of 2-PE guiding lines could be useful in directing termite activities away from or toward certain locations.

## Figures and Tables

**Table 1:** Results of field tests of 2-PE treatments applied to soil. Presence (“Termites”) or absence (-) of *C. formosanus* is indicated for each month after treatment.

Repetition	Field Stations	February	March	April	May	June	July	August
1	Control	-	-	-	-	-	-	-
	Treatment	-	-	-	-	-	-	-
2	Control	-	-	-	-	-	-	-
	Treatment	-	-	-	Termites	Termites	-	-
3	Control	-	-	-	-	-	-	-
	Treatment	-	-	-	-	Termites	-	-
4	Control	-	-	-	-	-	-	-
	Treatment	-	-	-	-	-	-	-
5	Control	-	-	-	-	-	-	-
	Treatment	-	-	-	-	-	-	-
6	Control	-	-	-	-	-	-	-
	Treatment	-	-	-	-	-	-	-
7	Control	-	-	-	-	-	-	-
	Treatment	-	-	-	-	-	-	-
8	Control	-	-	-	-	-	-	-
	Treatment	-	-	-	Termites	Termites	Termites	Termites
9	Control	-	-	-	-	-	-	-
	Treatment	-	-	-	-	Termites	Termites	Termites
10	Control	-	-	-	-		-	-
	Treatment	-	-	-	-	Termites	-	-

## Data Availability

All data is provided in the manuscript.

## References

[1] Becker G., Mannesmann R. (1968). Untersuchungen über das Verhalten von Termiten Gegenüber Einigen Spurbildenden Stoffen. Z. Angew. Entomol..

[2] Chen J., Henderson G., Laine R.A. (1998). Isolation and Identification of 2-Phenoxyethanol from a Ballpoint Pen Ink as a Trail-Following Substance of *Coptotermes formosanus* Shiraki and *Reticulitermes* sp.. J. Entomol. Sci..

[3] Cornelius M.L., Bland J.M. (2001). Trail-Following Behavior of *Coptotermes formosanus* and *Reticulitermes flavipes* (Isoptera: Rhinotermitidae)—Is There a Species-Specific Response?. Environ. Entomol..

[4] LaPorte G.M., Wilson J.D., Cantu A.A., Mancke S.A., Fortunato S.L. (2003). The Identification of 2-Phenoxyethanol in Ballpoint Inks Using Gas Chromatography/Mass Spectrometry—Relevance to Ink Dating. J. Forensic Sci..

[5] Howard R., Matsumura F., Coppel H.C. (1976). Trail-following Pheromones of the Rhinotermitidae: Approaches to Their Authentication and specificity. J. Chem. Ecol..

[6] Safety and Health (NIOSH) (1991). Criteria for a Recommended Standard—Occupational Exposure to Ethylene Glycol Monomethyl Ether, Ethylene Glycol Monoethyl Ether, and Their Acetates.

[7] Rastogi S.C. (2000). Analytical Control of Preservative Labelling on Skin Creams. Contact Dermat..

[8] Dréno B., Zuberbier T., Gelmetti C., Gontijo G., Marinovich M. (2019). Safety Review of Phenoxyethanol When Used as a Preservative in Cosmetics. J. Eur. Acad. Dermatol. Venereol..

[9] Tosti A., Vincenzi C., Trevisi P., Guerra L. (1995). Euxyl K 400: Incidence of Sensitization, Patch Test Concentration and Vehicle. Contact Dermat..

[10] Vogt T., Landthaler M., Stolz W. (1998). Generalized Eczema in an 18-month-old Boy Due to Phenoxyethanol in DPT Vaccine. Contact Dermat..

[11] Jordan W. (2013). New Concepts in Management of Drywood (Blattodea: Kalotermitidae) and Subterranean Termites (Blattodea: Rhinotermitidae). Ph.D. Dissertation.

[12] Schneider C.A., Rasband W.S., Eliceiri K.W. (2012). NIH Image to ImageJ: 25 years of image analysis. Nat. Methods.

[13] SAS Institute (2021). SAS Institute Inc. 2020–2021. JMP^®^ 16 Documentation Library.

[14] Fei H., Henderson G. (2005). Repellency of Formosan Subterranean Termites (Isoptera: Rhinotermitidae) to Dead Termites and Attraction to 2-phenoxyethanol with and without Nonrepellent Insecticides. J. Agric. Urban Entomol..

[15] Fei H., Henderson G., Fugler A.R., Laine A. (2005). Increased Search Tunnel Formation by *Coptotermes formosanus shiraki* (Isoptera: Rhinotermitidae) in 2-phenoxyethanol Treated Sand. J. Entomol. Sci..

[16] Su N.-Y., Scheffrahn R. (1990). Economically Important Termites in the United States and Their Control. Sociobiology.

[17] Janowiecki M.A., Austin J.W., Szalanski A.L., Vargo E.L. (2021). Identification of *Reticulitermes* Subterranean Termites (Blattodea: Rhinotermitidae) in the Eastern United States Using Inter-simple Sequence Repeats. J. Econ. Entomol..

[18] Matsumura F., Coppel H.C., Tai A. (1968). Isolation and Identification of Termite Trail-following Pheromone. Nature.

[19] Traniello J.F.A., Leuthold R.H., Abe T., Bignell D.E., Higashi M. (2000). Behavior and Ecology of Foraging in Termites. Termites: Evolution, Sociality, Symbioses, Ecology.

[20] Mizumoto N., Kobayashi K., Matsumura K. (2015). Emergence of intercolonial variation in Termite Shelter Tube Patterns and Prediction of its Underlying Mechanism. R. Soc. Open Sci..

[21] Puche H., Su N.-Y. (2001). Application of Fractal Analysis for Tunnel Systems of Subterranean Termites (Isoptera: Rhinotermitidae) Under Laboratory Conditions. Environ. Entomol..

[22] Haverty M. (1976). Termites. Pest Control.

[23] Cornelius M.L., Osbrink W.L.A. (2010). Effect of Soil Type and Moisture Availability on the Foraging Behavior of the Formosan Subterranean Termite (Isoptera: Rhinotermitidae). J. Econ. Entomol..

[24] Costa-Leonardo A.M. (2006). Morphology of the Sternal Gland in Workers of *Coptotermes gestroi* (Isoptera, Rhinotermitidae). Micron.

[25] Reinhard J., Kaib M. (2001). Trail Communication During Foraging and Recruitment in the Subterranean Termite *Reticulitermes santonensis* De Feytaud (Isoptera, Rhinotermitidae). J. Insect Behav..

[26] Ebeling W. (1978). Urban Entomology.

[27] Houseman R.M., Gold R.E. (2003). Factors that Influence Tunneling in the Eastern Subterranean Termite, *Reticulitermes flavipes* (Kollar) (Isoptera: Rhinotermitidae). J. Agric. Urban Entomol..

[28] Lee S.-H., Bardunias P., Su N.-Y. (2007). Optimal Length Distribution of Termite Tunnel Branches for Efficient Food Search and Resource Transportation. Biosystems.

[29] Su N.Y., Stith B.M., Puche H., Bardunias P. (2004). Characterization of Tunneling Geometry of Subterranean Termites (lsoptera: Rhinotermitidae) by Computer. Sociobiology.

[30] Esenther G.R., Allen T.C., Casida J.E., Shenefelt R.D. (1961). Termite Attractant from Fungus-Infected Wood. Science.

[31] Su N.-Y. (2005). Directional Change in Tunneling of Subterranean Termites (Isoptera: Rhinotermitidae) in Response to Decayed Wood Attractants. J. Econ. Entomol..

[32] Robson S.K., Lesniak M.G., Kothandapani R.V., Traniello J.F.A., Thorne B.L., Fourcassié V. (1995). Nonrandom search geometry in subterranean termites. Naturwissenschaften.

[33] Bardunias P.M., Su N.-Y. (2010). Queen Size Determines the Width of Tunnels in the Formosan Subterranean Termite (Isoptera: Rhinotermitidae). J. Insect Behav..

[34] Bruinsma O.H. (1979). An Analysis of Building Behaviour of the Termite *Macrotermes subhyalinus* (Rambur). Ph.D. Thesis.

[35] Grassé (1984). La Fondation des Nouvelles Sociétés. Termitolo…-Google Scholar. https://scholar.google.com/scholar_lookup?title=Termitologia%20%E2%80%93%20Tome%20II&author=PP.%20Grass%C3%A9&publication_year=1984.

[36] Kaur G.T., Gajalakshmi A.S., Abbasi S.A. (2018). Laboratory studies on trail following behavior of the termite *Hypotermes obscuriceps* towards 2-Phenoxyethanol. Int. J. Entomol. Res..

[37] Ibrahim S.A., Henderson G., Fei H., Laine R.A. (2005). 2-Phenoxyethanol is a doubleedged sword against the Formosan subterranean termite (Isoptera: Rhinotermitidae). Biopestic. Int..

[38] Mizumoto N., Bourguignon T. (2020). Modern termites inherited the potential of collective construction from their common ancestor. Ecol. Evol..

[39] Neoh K.-B., Yeap B.-K., Tsunoda K., Yoshimura T., Lee C.-Y. (2012). Do Termites Avoid Carcasses? Behavioral Responses Depend on the Nature of the Carcasses. PLoS ONE.

[40] Runcie C.D. (1987). Behavioral evidence for multicomponent trail pheromone in the termite, *Reticulitermes flavipes* (Kollar) (Isoptera: Rhinotermitidae). J. Chem. Ecol..

[41] Pasteels J., Roisin Y., Bourguignon T. (2007). Taxonomy, distribution and host specificity of the termitophile tribe Trichopseniini (Coleoptera: Staphylinidae) in New Guinea and adjacent islands. Insect Syst. Evol..

[42] Stuart A.M. (1961). Mechanism of Trail-laying in Two Species of Termites. Nature.

[43] Su N.Y., Bardunias P. (2005). Foraging behavior of subterranean termites (Isoptera: Rhinotermitidae): Food discovery and movement of termites within established galleries. Proceedings of the Fifth International Conference on Urban Pests, Singapore, 11–13 July 2005.

[44] Robert A., Peppuy A., Sémon E., Boyer F.D., Lacey M.J., Bordereau C. (2004). A new C12 alcohol identified as a sex pheromone and a trail-following pheromone in termites: The diene (Z,Z)-dodeca-3,6-dien-1-ol. Naturwissenschaften.

[45] Tucker C.L., Koehler P.G., Oi F.M. (2004). Influence of Soil Compaction on Tunnel Network Construction by the Eastern Subterranean Termite (Isoptera: Rhinotermitidae). J. Econ. Entomol..

